# Notoginsenoside R1 attenuates sevoflurane-induced neurotoxicity

**DOI:** 10.1515/tnsci-2020-0118

**Published:** 2020-06-22

**Authors:** Yibing Zhang, Yong Zhao, Yongwang Ran, Jianyou Guo, Haifeng Cui, Sha Liu

**Affiliations:** Comprehensive Teaching and Research Office of Traditional Chinese Medicine, School of Traditional Chinese Medicine, Chongqing Medical University, Chongqing, 401331, People’s Republic of China; GLP Laboratory, Institute of Chinese Materia Medica, China Academy of Traditional Chinese Medicine, Beijing, 100700, People’s Republic of China; Department of Radiology, Qianjiang Central Hospital of Chongqing, Chongqing, 409099, People’s Republic of China; Institute of Psychology, Chinese Academy of Sciences, Beijing, 100101, People’s Republic of China

**Keywords:** 5′-AMP-activated protein kinase, neurotoxicity, notoginsenoside R1, sevoflurane, sestrin-2, reactive oxygen species

## Abstract

**Background:**

Sevoflurane, a volatile anesthetic, is known to induce widespread neuronal degeneration and apoptosis. Recently, the stress-inducible protein sestrin 2 and adenosine monophosphate-activated protein kinase (AMPK) have been found to regulate the levels of intracellular reactive oxygen species (ROS) and suppress oxidative stress. Notoginsenoside R1 (NGR1), a saponin isolated from *Panax notoginseng*, has been shown to exert neuroprotective effects. The effects of NGR1 against neurotoxicity induced by sevoflurane were assessed.

**Methods:**

Sprague-Dawley rat pups on postnatal day 7 (PD7) were exposed to sevoflurane (3%) anesthesia for 6 h. NGR1 at doses of 12.5, 25, or 50 mg/kg body weight was orally administered to pups from PD2 to PD7.

**Results:**

Pretreatment with NGR1 attenuated sevoflurane-induced generation of ROS and reduced apoptotic cell counts. Western blotting revealed decreased cleaved caspase 3 and Bad and Bax pro-apoptotic protein expression. NGR1 substantially upregulated nuclear factor erythroid 2-related factor 2 (Nrf2) expression along with increased heme oxygenase-1 (HO-1) and NAD(P)H quinone oxidoreductase-1 levels, suggesting Nrf2 signaling activation. Enhanced sestrin-2 and phosphorylated AMPK expression were noticed following NGR1 pretreatment.

**Conclusion:**

This study revealed the neuroprotective effects of NGR1 through effective suppression of apoptosis and ROS via regulation of apoptotic proteins and activation of Nrf2/HO-1 and sestrin 2/AMPK signaling cascades.

## 1 Introduction

The inhalation anesthetic sevoflurane has a wide range of applications in clinical practice that demand surgery and pain management. Sevoflurane is an extensively used halogenated inhalation anesthetic in adults and as well in pediatric patients owing to its low pungency, non-irritating odor, low blood/gas ratio, and also due to immediate onset and recovery [1,2,3,4]. Nevertheless, several experimental data have reported that sevoflurane inhibits neurogenesis and induces neuronal apoptosis and neuroinflammation [5,6,7]. Excessive reactive oxygen species (ROS)-induced oxidative stress is said to be a significant causative factor in sevoflurane-induced neurotoxicity [8].

Sestrins, stress-inducible proteins, are known to be crucially involved in maintaining cell homeostasis and in cellular repair [9]. Accumulating experimental data have reported that sestrins shield cells under various stress conditions such as oxidative stress [10]. Sestrin-1, -2, and -3 are the three isoforms of sestrins expressed in mammalian cells. Sestrin-2 is a downstream effector of p53 that is crucially associated with the regulation of cell viability under several conditions of cellular stress [11,12]. Sestrin-2 was revealed to control intracellular levels of ROS and is critically involved in the regeneration of peroxiredoxins [11,12]. Sestrin-2 has also been shown to activate adenosine monophosphate-activated protein kinase (AMPK), which is also a stress-inducible protein that is pivotal in the regulation of cell growth, maintenance of metabolic homeostasis, and oxidative stress [9,13].

Nuclear factor erythroid 2-related factor 2 (Nrf2) is a key factor in transcription and is implicated in the regulation of oxidative stress conditions. Nrf2 regulates antioxidant enzymes and phase II enzymes involved in the detoxification process via antioxidant response element [14,15]. Enzymes, including glutathione *S*-transferases, heme oxygenase-1 (HO-1), and NAD(P)H quinone oxidoreductase-1 (NQO1), are regulated by Nrf2 [16,17].

Recently, much researchers have been concentrating on the protective effects of plant-derived compounds in brain injury [18,19]. *Panax notoginseng* (Burk.) F. H. Chen is a Chinese plant with extensive health benefits [20,21]. Notoginsenoside R1 (NGR1), a saponin extracted from *P. notoginseng*, has been reported to possess numerous beneficial properties including antioxidant [22], anti-inflammatory [23], cardioprotective [24], and neuroprotective [25]. Here, we explored the effects of systemic administration of NGR1 on neonatal rats prior to sevoflurane exposure.

## 2 Materials and methods

### 2.1 Chemicals

NGR1, sevoflurane, and all other chemicals were purchased from Sigma-Aldrich (St. Louis, MO, USA) unless otherwise specified. Antibodies against cleaved caspase-3, Bad, Bax, Bcl-2, Bcl-xL, Nrf2, HO-1, xIAP, cIAP-1, cIAP-2, and survivin were procured from Cell Signaling Technology (Beverly, MA, USA). AMPK, p-AMPK, and NQO1 were purchased from Santa Cruz Biotechnology (Santa Cruz, CA, USA). β-Actin and sestrin-2 (Abcam, USA) were used for protein expression analysis.

### 2.2 Animals

Timed-pregnant female Sprague-Dawley rats were provided by the Laboratory Animal Centre of Chongqing Medical University. The rats were accommodated in separate sterile cages under controlled conditions (22–23℃; 12:12 h light/dark cycle and 55–60% relative humidity). A standard laboratory rat pellet diet and drinking water were provided to the rats and were carefully observed for the time of delivery, and 1-week-old pups (weighing 50 ± 2 g; *n* = 72) were used for the study.

### 2.3 Ethical approval

The study design and its protocols were approved by the Animal Ethical Committee of the Chongqing Medical University and were in compliance with the National Institutes of Health (NIH) Guidelines for the Care and Use of Laboratory Animals (NIH publication no. 85-23, revised 1996). Efforts were taken to reduce animal suffering during the entire period of study [26].

### 2.4 Animal grouping and anesthetic exposure

The pups were divided into different study groups (*n* = 12 per group). The separate groups of pups were administered NGR1 (12.5, 25, or 50 mg/kg b.wt) every day from postnatal day 2 (PD2) until postnatal day 7 (PD7). On PD7, sevoflurane (3%) was given to the pups for 6 h in 70% air or oxygen [27]. The temperature was maintained between 33 and 35℃ throughout the period of sevoflurane exposure. NGR1 was administered 1 h prior to sevoflurane exposure on PD7. Control pups received no NGR1 or sevoflurane anesthesia; pups exposed to sevoflurane alone were treated as anesthetic controls and 12 h following anesthetic exposure, PD7 rat pups of different study groups were sacrificed. Instantly, brain tissues are excised and used for examination.

### 2.5 TUNEL assay

Sevoflurane anesthetic-induced neuroapoptosis was measured by the TUNEL assay. Hippocampal sections of 5 µm thickness were made and subjected to analysis using a DeadEnd TM fluorometric TUNEL system kit (Promega, Madison, WI, USA). Positive TUNEL cells were counted in the hippocampal CA1, CA3, and dentate gyrus (DG) regions of the brain that measure neuronal apoptosis. Positive TUNEL cells were observed and analyzed using an image processing system (NIS-Elements BR imaging processing and analysis software; Nikon Corporation, Japan).

### 2.6 Fluoro-Jade B (FJB) staining

Neurodegeneration after exposure to sevoflurane was assessed by FJB staining in hippocampal tissues. Hippocampal sections (30 μm thickness) affixed to gelatin-coated slides were dried overnight at room temperature, rehydrated, and incubated for 15 min in potassium permanganate solution (0.06%). The sections were rinsed well with distilled H_2_O and treated with FJB. After 30 min of incubation with 0.1% acetic acid, the slides were then observed and analyzed as mentioned in the previous section.

### 2.7 Determination of intracellular ROS

The hippocampal tissues were homogenized in phosphate-buffered saline (25 mg/mL) and centrifuged at 10,000 *g* for 5 min. The collected supernatant was used for analysis of intracellular ROS levels, lipid peroxidation, and antioxidant, glutathione (GSH) content. Total protein content in the collected supernatant was assessed using the Bradford protein assay kit (BioRad, Hercules, CA, USA). ROS generation was determined using the OxiSelect™ *in vitro* ROS/RNS assay kit (Green Fluorescence; Cell Bio Labs Inc.). A fluorogenic probe, dichlorodihydrofluorescein DiOxyQ (DCFH-DiOxyQ), specific for ROS/reactive nitrogen species (RNS), was used. DCFH is oxidized rapidly to a highly fluorescent 2′,7′-dichlorodihydrofluorescein (DCF) compound upon reaction with ROS/RNS. The intensity of the DCF formed was measured as the fluorescence intensity that was read using a SpectraMa-Gemini XPS microplate reader (Molecular Devices) at a wavelength of 480 nm excitation/530 nm emission.

### 2.8 Determination of lipid peroxidation and GSH levels

Lipid peroxidation levels following sevoflurane exposure were determined as malondialdehyde (MDA) content. MDA and GSH levels were measured as guided by the manufacturer using kits from Sigma-Aldrich.

### 2.9 Immunoblotting

Excised brain tissues (*n* = 6 per group) were homogenized using cell lysis buffer from Cell Signaling Technology, USA (20 mM Tris–HCl [pH 7.5], 1% Triton, 1 mM EGTA, 2.5 mM sodium pyrophosphate, 150 mM NaCl, 1 µg/mL leupeptin, 1 mM Na_3_VO_4_, 1 mM Na_2_EDTA, and 1 mM beta-glycerophosphate). The whole cell extracts obtained were subjected to centrifugation (3,000 rpm, 15 min) at 4℃. The supernatant collected was used for analysis. Cytosol and nuclear fractions were separated from the whole cell homogenate using ReadyPrep™ Protein Extraction Kit (Cytoplasmic/Nuclear) from Bio-Rad (CA, USA). Total protein contents of whole cell extracts and in the cytosol and nuclear fractions were quantified using kits from Thermo Fischer Scientific. For protein expression analysis, 60 μg amount of protein sample (*n* = 6 per group; Nrf2 in the nuclear fraction and HO-1 in the cytosolic fraction) was loaded and separated electrophoretically on sodium dodecyl sulfate-polyacrylamide gel electrophoresis (8–12%). The separated protein bands were blot transferred onto polyvinylidene fluoride (PVDF; Thermo Fischer Scientific), and the membranes were blocked in 5% non-fat dry milk in 1× TBST to exclude any endogenous peroxidase activity. After washing with TBST, the PVDF membranes were incubated overnight at 4°C with the test protein-specific primary antibodies. Following this, the membranes were further washed with TBST and treated with secondary antibody (horse radish peroxidase-conjugated; 1:2,000; Santa Cruz Biotechnology) at room temperature for 60 min. The membranes were rinsed again with TBST, and the bands were visualized using an enhanced chemiluminescence system (Millipore, USA). The positive bands obtained were scanned and detected using Image J software (Supersignal; Pierce, IL, USA).

### 2.10 Statistical study

The data obtained from the experiments were statistically analyzed using IBM SPSS software (version 21.0; SPSS Inc., Chicago, IL, USA). The values were subjected to analysis by one-way analysis of variance (one-way ANOVA) and Duncan’s multiple range test (DMRT) for comparison of data from multiple groups. Values at *p* < 0.05 were marked as statistically significant.

## 3 Results

### 3.1 NGR1 lowered sevoflurane-induced neuronal cell apoptosis

**Figure 1:**
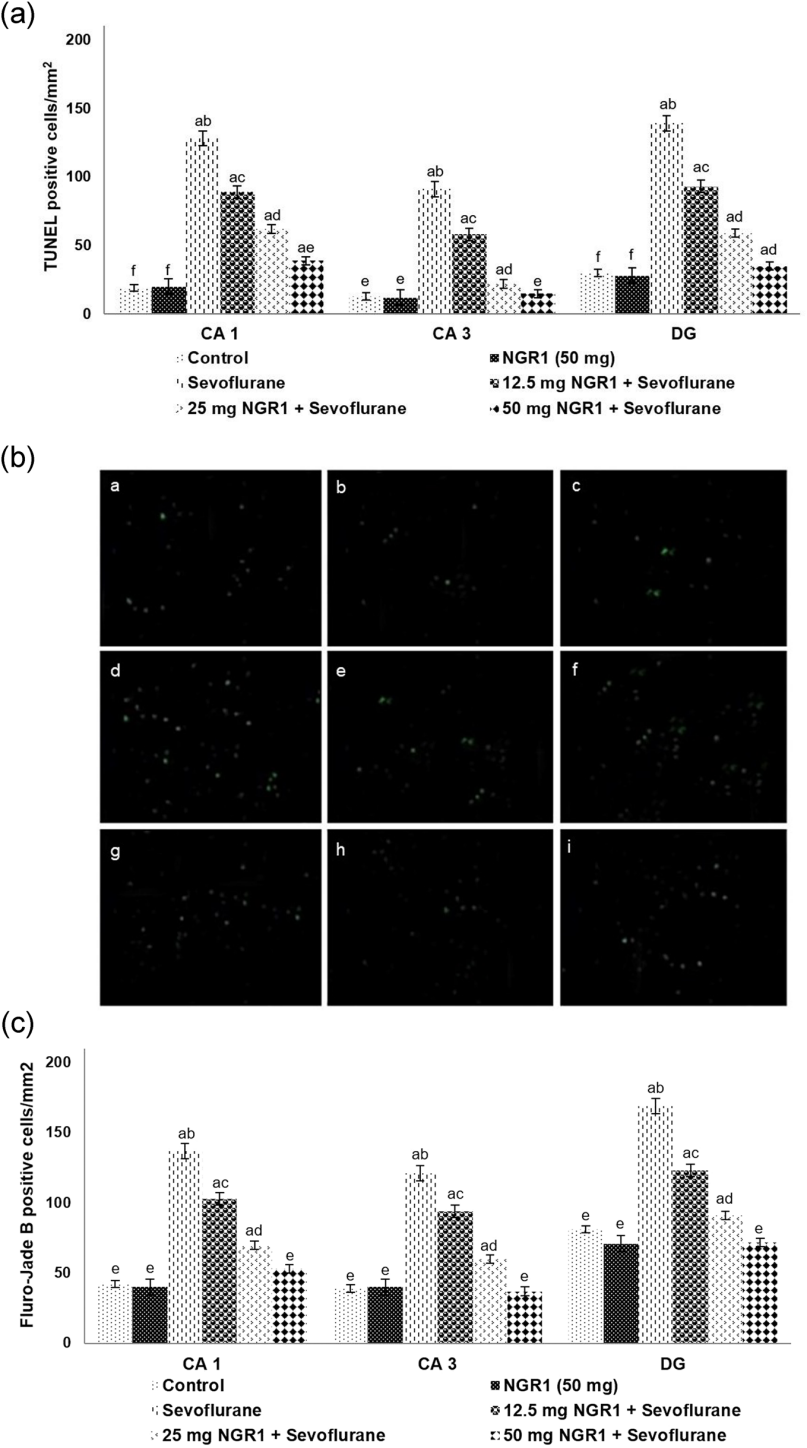
Influence of NGR1 on sevoflurane-induced neuroapoptosis. TUNEL-positive cells (a and b) and FJB-positive cells (c). Values are represented as mean ± SD, *n* = 6. *p* < 0.05 as determined by one-way ANOVA followed by DMRT analysis: a represents *p* < 0.05 vs control; b–f represent mean values from different experimental groups that at *p* < 0.05 (row: (a–c) NGR1 (50 mg); (d–f) sevoflurane; (g–i) 50 mg NGR1 + sevoflurane and column: (a, d, and g) CA1; (b, e, and h) CA3; and (c, f, and i) DG).

Sevoflurane exposure for 6 h resulted in a significant (*p* < 0.05) apoptosis in the hippocampal CA1, CA3, and in the DG regions (Figure 1a). NGR1 administration at 12.5, 25, and 50 mg/kg reduced (*p* < 0.05) TUNEL-positive cell counts effectively. TUNEL-positive cell counts were reduced to 39, 15, and 35 cells/mm^2^ in the CA1, CA3, and DG regions, respectively, in pups treated with 50 mg NGR1. FJB staining also exhibited a significant (*p* < 0.05) reduction in apoptotic cell number in pups that were administered NGR1 vs sevoflurane control (Figure 1b). A 50 mg dose of NGR1 was more effective in reducing apoptotic counts, as observed in the TUNEL assay and as well in FJB staining.

**Figure 2:**
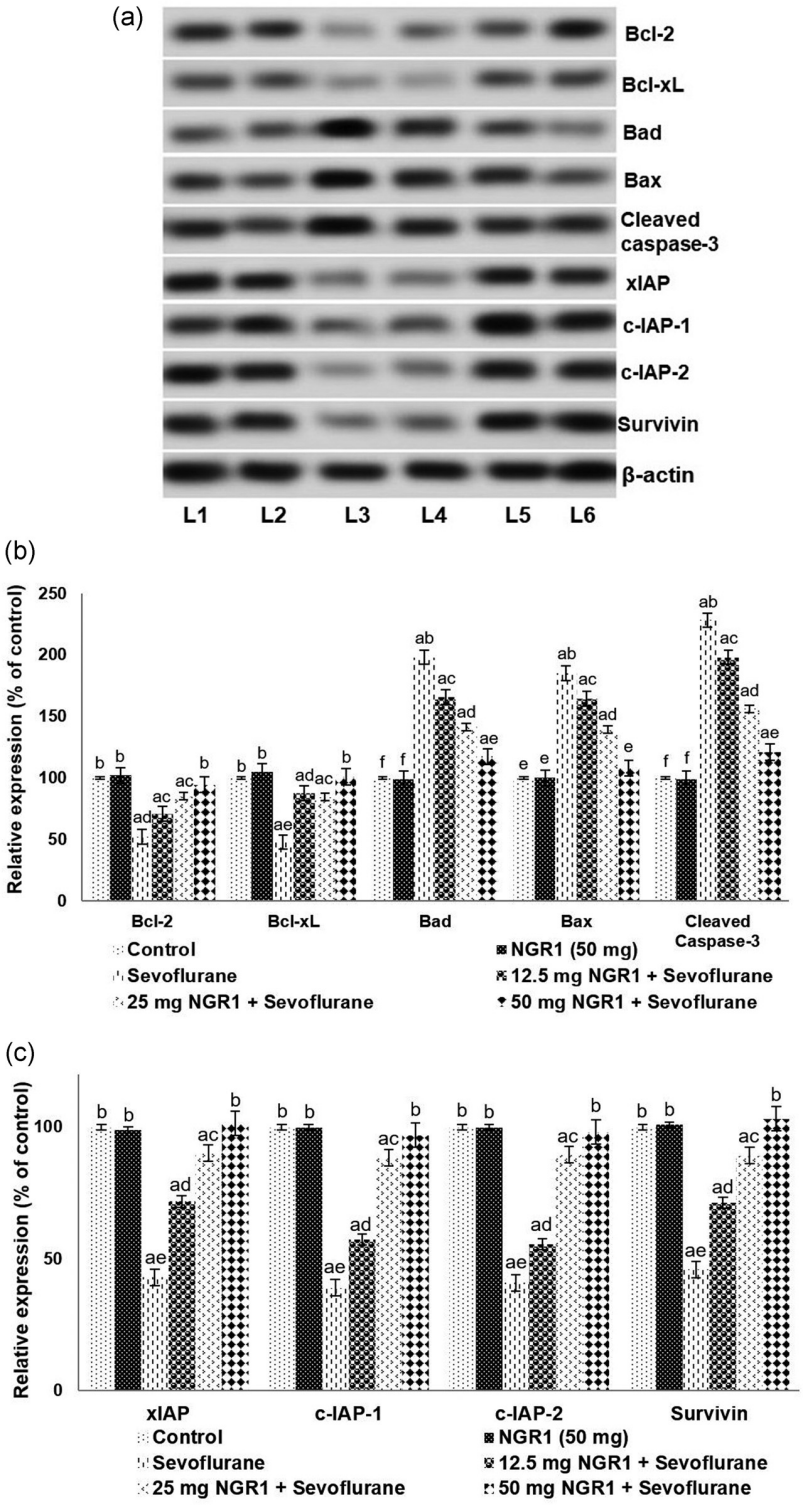
NGR1 modulates the expression of apoptosis pathway proteins (a–c). Values are represented as mean ± SD, *n* = 6. *p* < 0.05 as determined by one-way ANOVA followed by DMRT analysis. Relative expressions of proteins from different treatment groups with control expressions set at 100%: a represents *p* < 0.05 vs control; b–f represent mean values from various treatment groups that differ from each other at *p* < 0.05 L1-control; L2-NGR1 (50 mg); L3-sevoflurane; L4-12.5 mg NGR1 + sevoflurane; L5–25 mg NGR1 + sevoflurane; and L6-50 mg NGR1 + sevoflurane.

Immunoblotting studies revealed markedly (*p* < 0.05) enhanced activated caspase-3 expression (228.19%) in the PD7 pups exposed to sevoflurane vs normal control (Figure 2b). The data obtained illustrated that prior administration of NGR1 produced a significant (*p* < 0.05) decline in cleaved caspase-3 expression. The levels decreased from 228.19% to 198%, 156.15%, and 121.02% after treatment with 12.5, 25, and 50 mg NGR1.

The influence of NGR1 on the expression of proteins in the apoptotic cascade was also assessed. In this study, 6 h of exposure to sevoflurane caused a substantial (*p* < 0.05) elevation in the expression levels of pro-apoptotic proteins Bad (198%) and Bax (185.15%) in anesthetic control pups vs normal control (Figure 2a and b). Sevoflurane however caused a significant (*p* < 0.05) downregulation in anti-apoptotic protein expression. Bcl-xL levels decreased to 47.71% vs 100% in the normal control, while Bcl-2 decreased to 52%.

Furthermore, in line with depressed Bcl-xL and Bcl-2 levels, a multi-fold reduction (*p* < 0.05) in the expression of IAPs (xIAP, c-IAP-1, and c-IAP-2; inhibitors of apoptosis proteins) and survivin was noticed after sevoflurane exposure (Figure 2a and c). Pretreatment with NGR1 at the evaluated doses markedly (*p* < 0.05) upregulated Bcl-2 and Bcl-xL expression and levels of c-IAP-1, c-IAP-2, xIAP, and survivin. Interestingly, administration of NGR1 prior to sevoflurane induced a reduction in the expression of Bad and Bax levels and activated caspase-3. Thus, NGR1-mediated regulation of apoptotic protein expression could have aided in the decreased apoptotic cell counts, as noticed by FJB staining and the TUNEL assay. The results thus reveal the anti-apoptotic efficiency of NGR1.

### 3.2 NGR1 decreased sevoflurane-induced oxidative stress

**Figure 3:**
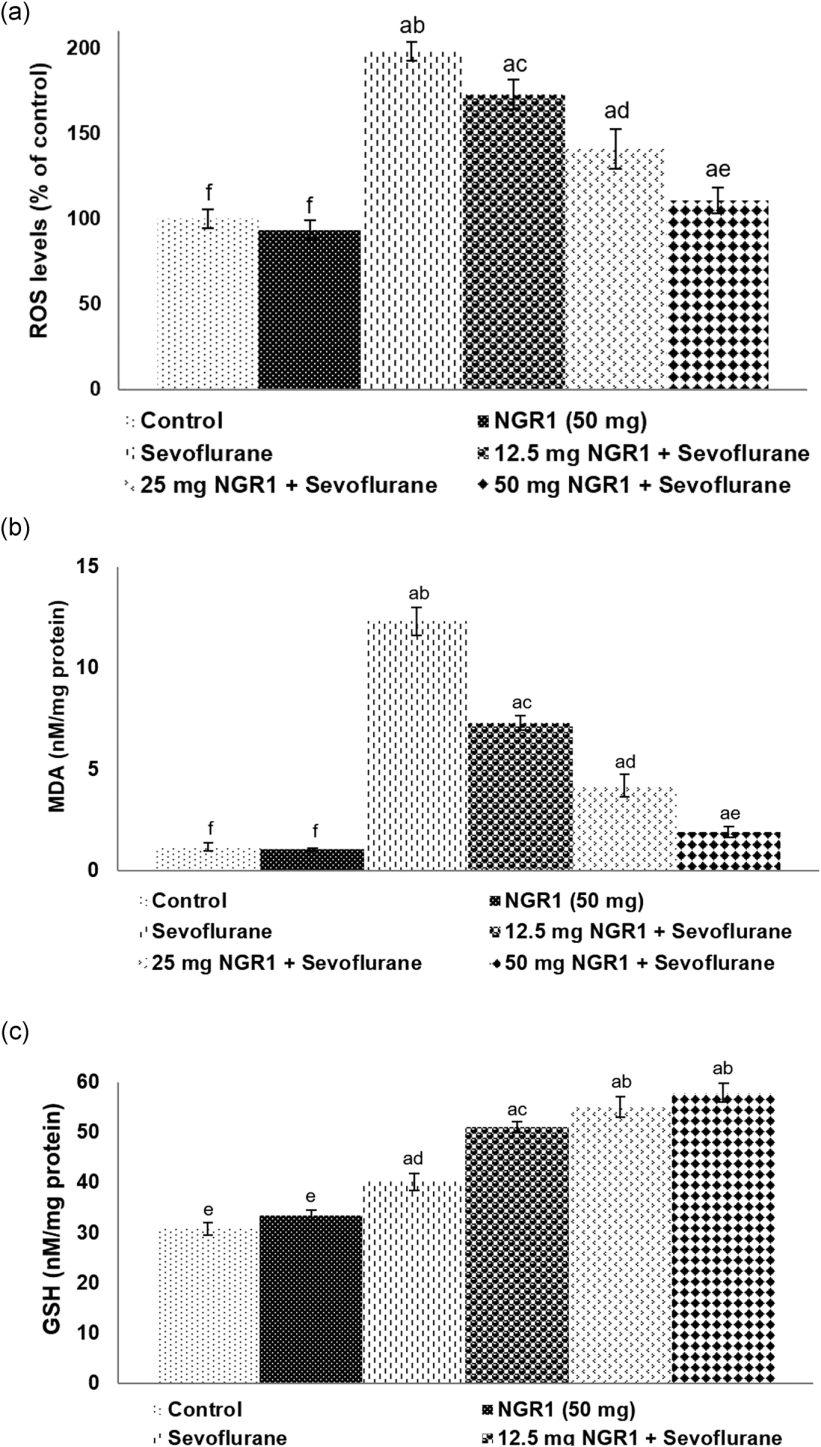
Influence of NGR1 on sevoflurane-induced oxidative stress. NGR1 reduced ROS levels (a) and MDA levels (b) and improved GSH levels (c). Values are represented as mean ± SD, *n* = 6: a represents *p* < 0.05 vs control; a–e represent mean values from different experimental groups that differ from each other at *p* < 0.05.

Sevoflurane exposure resulted in a multi-fold increase in ROS and MDA levels (Figure 3a and b). ROS generation was found to increase to 198.08% in PD7 pups exposed to sevoflurane alone vs normal control pups. The MDA content was observed to increase to 12.31 nM/mg protein at 12 h following sevoflurane exposure vs 1.16 nM/mg protein in normal control. Treatment with NGR1 prior to exposure to sevoflurane considerably (*p* < 0.05) reduced ROS generation and lipid peroxidation levels. ROS generation was reduced to 110.9% after 50 mg NGR1 treatment. The MDA levels were found to be significantly lower (*p* < 0.05) in pups pretreated with NGR1 vs anesthetic control pups. In 50 mg NGR1-treated pups exposed to sevoflurane presented MDA levels (1.89 nM/mg protein) close to normal control levels.

Interestingly, the levels of antioxidant, GSH in the anesthetic control rats were noticed to be significantly (*p* < 0.05) increased 12 h post sevoflurane exposure. The GSH content increased to 40.19 nM/mg protein in anesthetic control pups vs 30.76 nM/mg protein in the normal control (Figure 3c). Systemic administration of NGR1 prior to sevoflurane exposure from PD2 to PD7 resulted in a markedly increased GSH content in the brain tissues. The levels increased to 51.03, 55.1, and 57.89 nM/mg protein on treatment with 12.5, 25, and 50 mg NGR1, respectively. Furthermore, rats that were treated with 50 mg NGR1 alone presented GSH levels of 33.5 nM/mg protein and the levels were comparable to that of the normal control.

### 3.3 NGR1 promoted Nrf2 signaling

**Figure 4:**
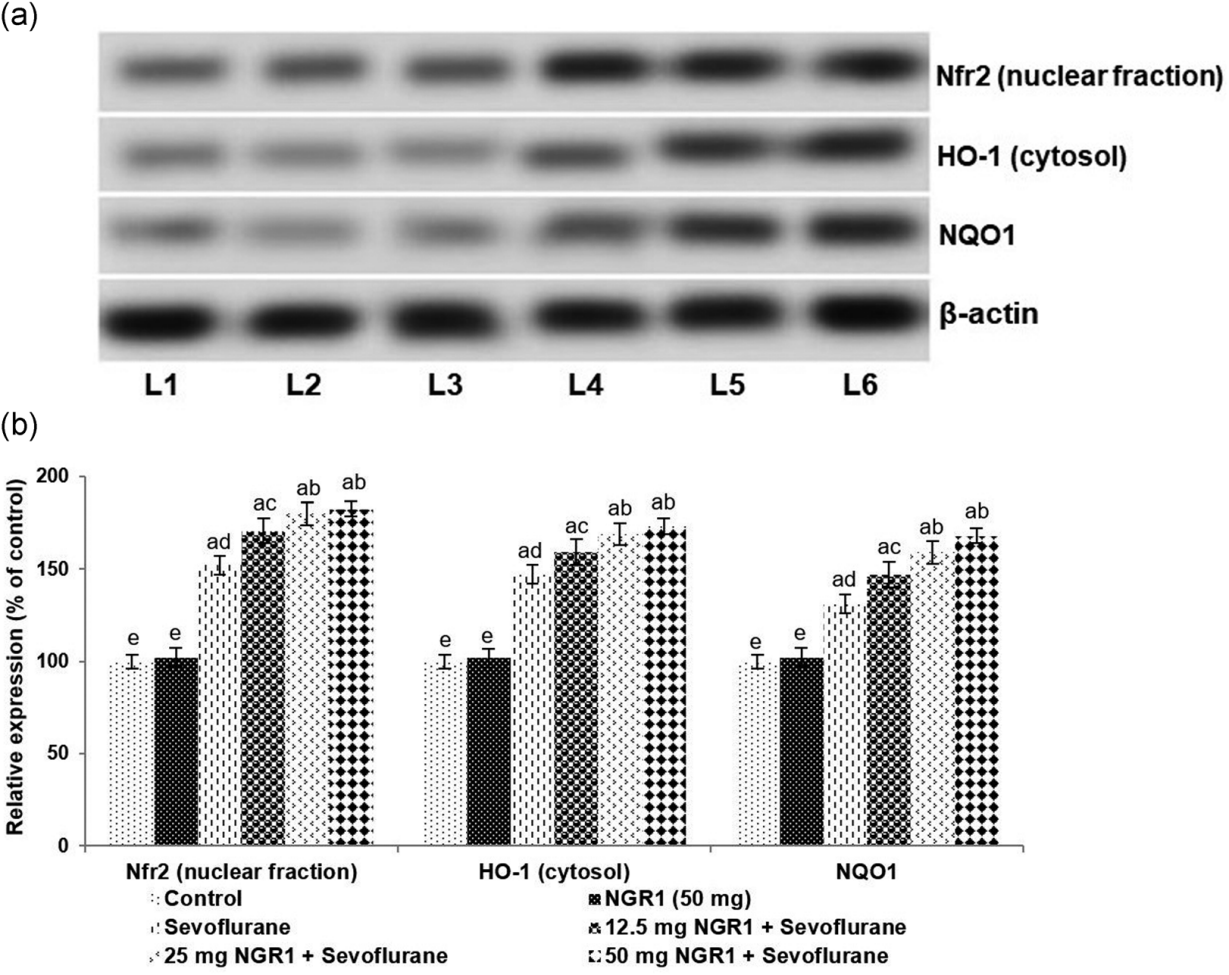
NGR1 regulates the antioxidant regulators. (a) Representative immunoblot of protein expressions. (b) Relative expressions of test proteins. Values are represented as mean ± SD, *n* = 6. *p* < 0.05 as determined by one-way ANOVA followed by DMRT analysis. Relative expressions of proteins from different treatment groups with control expressions set at 100%: a represents *p* < 0.05 vs control; b–f represent mean values from various treatment groups that differ at *p* < 0.05 L1-control; L2-NGR1 (50 mg); L3-sevoflurane; L4-12.5 mg NGR1 + sevoflurane; L5–25 mg NGR1 + sevoflurane; and L6–50 mg NGR1 + sevoflurane.

The expression of Nrf2, NQO1, and HO-1 was assessed following 12 h post sevoflurane exposure by immunoblotting. The observed results indicated that systemic administration of NGR1 at all the three tested doses caused a significant (*p* < 0.05) upregulation in Nrf2, HO-1, and NQO1 expression (Figure 4a and b). While the NQO1 and HO-1 expression increased to 131.01% and 146.99% in anesthetic control pups compared to normal control. Prior treatment with NGR1 enhanced nuclear expression of Nrf2 along with elevated NQO1 and HO-1 in the cytosol. The expression of Nrf2 was upregulated to 182.4% upon treatment with 50 mg NGR1. The upregulated expression of Nrf2 suggests the activation of the pathway by NGR1.

### 3.4 Sestrin-2 expression is regulated by NGR1

**Figure 5:**
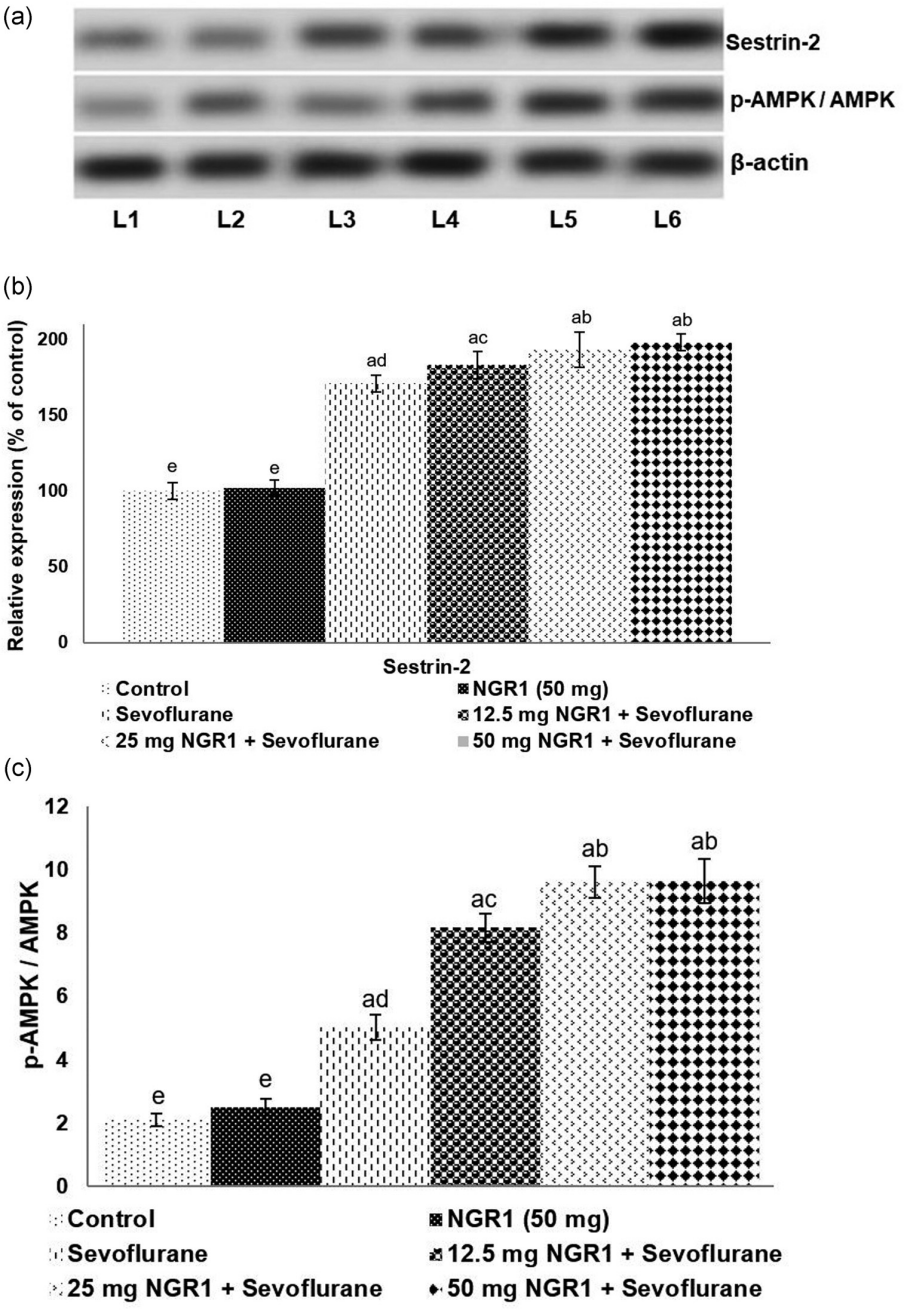
NGR1 activates sestrin 2 signaling. (a) Representative immunoblot of sestrin-2 (b) and p-AMPK/AMPK ratio (c). Values are represented as mean ± SD, *n* = 6. *p* < 0.05 as determined by one-way ANOVA followed by DMRT analysis. Relative expressions of proteins from various treatment groups with control expressions set at 100%: a represents *p* < 0.05 vs control; b–e represent mean values from different study groups that differ at *p* < 0.05. L1-control; L2-NGR1 (50 mg); L3-sevoflurane; L4-12.5 mg NGR1 + sevoflurane; L5–25 mg NGR1 + sevoflurane; and L6–50 mg NGR1 + sevoflurane.

Sevoflurane-induced stress produced a significant (*p* < 0.05) increase in sestrin-2 expression (Figure 5a and b). NGR1 administration from PD2 to PD7 prior to sevoflurane on PD7 resulted in a substantial (*p* < 0.05) upregulation in the expression of sestrin-2 vs normal control. The expression of phosphorylated AMPK was also noticed to be enhanced significantly (*p* < 0.05) in line with sestrin-2 expression, indicating activation of AMPK. These enhanced expressions suggest activation of sestrin-2/AMPK signaling under sevoflurane-induced stress. Furthermore, NGR1 administration was also found to upregulate sestrin-2 expression and as well enhance phosphorylation of AMPK. Sestrin-2 expression increased to 198% on NGR1 (50 mg) vs 171% in anesthetic control. These results indicate the efficacy of NGR1 to induce sestrin-2 signaling to combat sevoflurane-induced neurotoxicity.

## 4 Discussion

Sevoflurane, one of the extensively used volatile anesthetics, is known to cause severe neuronal apoptosis and long-term memory and cognitive deficits [27,28,29]. Studies on nerve cells have reported that sevoflurane induces overproduction of ROS [8]. The effects of oral administration of NGR1 to rat pups that were exposed to sevoflurane-induced neurotoxicity were analyzed in this study. PD7 rat pups were taken for the present investigation based on the previous experimental data that suggested that PD6–PD10 is the most crucial period of neurogenesis and brain development in rodents, rats, and mice. Furthermore, it has been observed to be the most susceptible time period to cell damage by external insult such as anesthesia [30,31].

Furthermore, sevoflurane-induced developmental neurotoxicity was found to positively correlate with the extent of hippocampal injury [32]. In this study, we assessed neuronal apoptosis in the hippocampal tissues 12 h post sevoflurane treatment by TUNEL and FJB staining. Severe apoptosis was noticed in the hippocampal CA1, CA3, and DG regions of PD7 rat pups after 6 h of sevoflurane exposure. Studies have shown that sevoflurane-induced neuronal degeneration encompasses the caspase pathway activation via the Bax-dependent intrinsic apoptosis pathway, which subsequently causes cytochrome *c* translocation from the mitochondria [28,33].

Caspase 3, a cysteine protease, is an executioner caspase enzyme. Activation of caspase-3 results in DNA degradation and the formation of apoptotic bodies [34,35]. It has been shown that exposure to anesthetics accelerates cellular apoptosis via upregulation of Bax and activation of caspase 3 [36,37,38]. Cleaved caspase-3 expression is regarded the reference point for anesthetic-induced cell death by apoptosis [39,40]. Here, western blot studies have shown that sevoflurane induces cleaved caspase-3 expression, in line with previous studies [27,37]. Furthermore, marked upregulation of Bad and Bax proteins with downregulated levels of Bcl-xL and Bcl-2 after 6 h of sevoflurane indicates activation of the apoptotic pathway.

Under numerous apoptotic stimuli, Bax (pro-apoptotic protein) is found to translocate to mitochondria from the cytosol. This translocation subsequently results in cytochrome *c* release to the cytosol from mitochondria, which causes the activation of the caspases and thus induces the apoptotic cascade [41]. Bcl-2 and Bcl-xL proteins prevent the translocation of cytochrome *c* by blocking Bax, maintaining the mitochondrial membrane potential [42] and inhibiting apoptosis. Prior treatment with NGR1 significantly decreased cleaved caspase-3 expression. NGR1-mediated downregulation of Bax and Bad, along with raised expression of Bcl-2 and Bcl-xL, reflects the inhibition of the caspase cascade. NGR1 upregulated the expression of survivin and xIAP, cIAP-1, and cIAP-2. The upregulation of cell survival proteins and anti-apoptotic factors in part could have aided in decreased TUNEL and FJB-positive cell counts.

Elevated ROS and lipid peroxidation levels observed in the study after sevoflurane exposure are suggestive of oxidative stress. Previous studies have reported oxidative stress as a contributing factor in neuronal death induced by sevoflurane [8,43]. NGR1 treatment effectively inhibited MDA and ROS and enhanced GSH levels dose-dependently.

The expression of proteins Nrf2, NQO1, and HO-1 was also found to be significantly (*p* < 0.05) increased at 12 h following sevoflurane exposure. Nrf2, a major transcription factor, is a chief regulator of innate antioxidative responses in the brain. This elevation reflects the stimulation of innate defense mechanisms under oxidative stress conditions. Elevated HO-1 expression is reported to decrease ROS generation and as well significantly reduce cell membrane damage and also prevent neuronal degeneration [44,45]. NGR1 administration prior to sevoflurane caused a significant increase in Nrf2 and HO-1 expression. The increased Nrf2 and HO-1 expression was found to be in line with decreased ROS and MDA levels. The results illustrate that the NGR1-mediated decrease in ROS levels could be due to its direct antioxidant effects or an increase in Nrf2/HO-1 signaling. These observations indicate the efficacy of NGR1.

Furthermore, the results revealed markedly increased sestrin-2 expression following sevoflurane-induced oxidative stress. Also, a substantially increased phosphorylation of AMPK was noted in line with increased sestrin-2 levels. The upregulation of sestrin-2 expression noted that post sevoflurane exposure could be considered a cellular defense response under stress stimuli. Sestrins have been shown to protect cells under oxidative stress [10]. Sestrin-2 is a stress-inducible protein present in neuronal cells and is an intrinsic antioxidant factor [8,10,46]. AMPK is a serine/threonine protein kinase that controls and regulates cellular ATP levels by maintaining the AMP-ATP ratio. AMPK is activated under conditions that lead to the depletion of cellular ATP levels, such as glucose deprivation, oxidative stress, hypoxia, and ischemia [10,47]. Sestrin-2 activates AMPK by stimulating phosphorylation of AMPK [10]. Thus, enhanced p-AMPK levels could be attributed to elevated sestrin 2 expression.

NGR1 pretreatment prior to sevoflurane exposure was found to further significantly enhance sestrin 2 and p-AMPK expression in comparison to the sevoflurane alone exposed group, which demonstrates that NGR1 also aids the intrinsic defense factors under oxidative stress conditions apart from its direct antioxidant effects.

## 5 Conclusion

The observed data demonstrate that pretreatment with NGR1 potentially reduced sevoflurane-induced apoptosis and oxidative stress. NGR1 effectively reduced apoptosis and oxidative stress levels by modulating the expression of the proteins of cellular apoptosis and by effectively upregulating the Nrf2/HO-1 pathway and sestrin 2/AMPK signaling. These findings reveal the efficacy of NGR1 against sevoflurane-induced neurotoxicity.
